# Draft Genome Sequence of *Mycobacteroides* sp. Strain LB1, Isolated from the Sputum of a Cystic Fibrosis Patient

**DOI:** 10.1128/MRA.00797-20

**Published:** 2020-11-19

**Authors:** Peter Menzel, Rolf Schwarzer, Johannes Helmuth, Inna Friesen

**Affiliations:** a Labor Berlin-Charité Vivantes GmbH, Berlin, Germany; Queens College

## Abstract

We report the draft genome sequence of *Mycobacteroides* sp. strain LB1, isolated from the sputum of a cystic fibrosis patient in Berlin, Germany. The genome size is 4.9 Mbp with a GC content of 63.8%. The genome is only distantly related to other *Mycobacteroides* species, suggesting that it may represent a novel species.

## ANNOUNCEMENT

*Mycobacteroides* is a recently proposed bacterial genus in the family *Mycobacteriaceae*, comprising rapid-growing species in the former Mycobacterium abscessus/M. chelonae cluster (1). While *Mycobacteroides* species typically inhabit natural environments, such as soil or water, some species are opportunistic human pathogens that can cause infections in a variety of organs (2). The risk of infection is typically increased by underlying conditions, such as depression of the immune system or lung diseases, e.g., cystic fibrosis (3). Here, we report the genome of a possibly novel species, *Mycobacteroides* sp. strain LB1, which was isolated from the sputum of a 22-year-old patient from Berlin (Germany) with the underlying conditions of cystic fibrosis and severe bronchiectasis.

Cultivation of the isolate from an undiluted and decontaminated sample on Middlebrook 7H9 broth under aerobic conditions was successful after 21 days in a Bactec MGIT 960 culture system (Becton Dickinson) at 30°C. No growth on solid media (Löwenstein-Jensen and Stonebrink agars, Artelt-Enclit) could be obtained after subcultivation with incubation for 16 weeks. Due to the failed growth during subcultivation, antibiotic susceptibility testing could not be performed. The tuberculosis antigen MPT64 rapid test (Becton Dickinson) for detection of the presence of the Mycobacterium tuberculosis complex was negative. Thereafter, species identification was performed using the GenoType *Mycobacterium* common mycobacteria (CM) assay v2.0 (Hain Lifescience GmbH), which identified the isolate as an unspecified *Mycobacterium* species. Further, sequencing of the V3-V4 region of the 16S rRNA gene, using the Illumina 16S metagenomic sequencing protocol (https://support.illumina.com/content/dam/illumina-support/documents/documentation/chemistry_documentation/16s/16s-metagenomic-library-prep-guide-15044223-b.pdf) and 2 × 250-bp reads on the Illumina MiSeq platform, also could not resolve the species, and we therefore opted for whole-genome sequencing.

Genomic DNA was extracted from the liquid culture (see above) and purified using the DNeasy PowerSoil pro kit (Qiagen), and an Illumina sequencing library was prepared using the QiaSeq FX kit (Qiagen) following the manufacturer’s protocol. Sequencing was performed on an Illumina MiSeq platform using 2 × 250-bp paired-end reads, resulting in 1.5 million read pairs. After adapter trimming by fastp v0.20.0 (4), reads were assembled *de novo* with the assembly pipeline shovill v1.0.9 (https://github.com/tseemann/shovill), which uses SPAdes v3.14.0 (5). The assembly comprises 16 contigs longer than 500 bp, with a combined length of 4.9 Mbp and an *N*_50_ value of 596 kbp. The GC content is 63.8%. Default parameters were used for all programs.

The assembly was screened against all complete and draft bacterial genomes available in the NCBI RefSeq database (6) on 30 June 2020 using Mash Screen v2.0 (7). Genome-wide average nucleotide identities (ANI) between the identified most similar genomes and our assembly were calculated using FastANI v1.3 (8). The genomes most similar to that of *Mycobacteroides* sp. strain LB1 belong to several strains of Mycobacteroides salmoniphilum, with a maximum ANI of 85.9%. Other *Mycobacteroides* species show lower similarities, such as M. chelonae (84.5% ANI), M. saopaulense (84.3% ANI), and M. franklinii (84.2% ANI), and the most similar species from a different genus is Mycolicibacterium fortuitum (78.0% ANI). The *Mycobacteroides* sp. strain LB1 genome was annotated via the NCBI Prokaryotic Genome Annotation Pipeline (9), which identified 4,659 protein coding sequences.

Given the atypical slow growth and low genome similarity to other *Mycobacteroides* species, *Mycobacteroides* sp. strain LB1 possibly represents a novel species within this genus.

### Data availability.

Sequencing data are available at NCBI BioProject PRJNA644770, and the genome assembly is available at DDBJ/ENA/GenBank under the accession number JACCFF000000000.
